# Personalised treatment for older adults with cancer: The role of frailty assessment

**DOI:** 10.1016/j.tipsro.2020.09.001

**Published:** 2020-10-17

**Authors:** Anita O'Donovan, Michelle Leech

**Affiliations:** University of Dublin Trinity College, Dublin 8, Ireland

**Keywords:** Frailty, Radiotherapy, Older person, Cancer, Comprehensive geriatric assessment

## Abstract

It is widely accepted in clinical practice that chronological age is a poor predictor of treatment tolerance and outcomes in older adults with cancer. Intrinsic vulnerability is more a function of underlying frailty, rather than chronological age. Frailty is a state of increased vulnerability to stressors, such as cancer and its treatment, which can lead to adverse health outcomes for patients. Capturing this heterogeneity in reserve capacity is the cornerstone of management in geriatricmedicine, but remains poorly understood or adopted in radiation oncology. A two-step approach, using a shorter screening tool, followed by full assessment for those who need it, is the mostresourceful way of implementing frailty assessment in radiotherapy departments. It is important for radiation oncology professionals to identify frailty and to use this information in multidisciplinary decision making in order to develop a personalised radiotherapy approach for the older person. There are many ways we can effectively use this information, such as considering treatment fractionation schedules that would limit the burden of travel for those with social frailty, or reviewing the range of modalities at our disposal, which might limit toxicity in the older person at high risk of deterioration during treatment. Frailty assessment is not carried out in many radiotherapy departments presently, but there are many international models to use as exemplars as to how it may be implemented in clinical practice. There are many opportunities for further research and role development in this field at the current time.

## Highlights

• Treatment decisions in older adults receiving radiotherapy is often difficult.
• Older people with cancer are significantly under-represented in trials.
• This is especially true of frailer older adults.
• Personalised radiotherapy should include frailty assessment.

## Introduction

Making treatment decisions for those who are older often proves difficult, as there is a significant lapse in evidence-based radiation oncology. Older adults are under-represented in clinical trials [1], [2], [3], [4], despite the incidence of cancer in this age group, estimated to be 60% of all cancer cases [5]. Studies have highlighted the lack of adherence to standard guidelines for older adults [6], although, in reality, guidelines are limited, due to the aforementioned lack of trials in older age groups.

This lack of empirical evidence, and resultant difficulties in making treatment decisions may result in under-treatment, or indeed overtreatment. Taking the most commonly diagnosed cancer in men, prostate cancer, as an example, one study in locally advanced disease, found that the likelihood of patients receiving radical treatment was more than halved with every 10 year age increase [7]. Similarly, Yang et al. (2017) examined the receipt of definitive treatments, including radiotherapy, in intermediate and high-risk older patients with prostate cancer [8]. Age stratification revealed that 83% of those aged 75–79, and only 63% aged ≥80, intermediate-risk patients, received definitive therapy, while 81% aged 70–75 and 55% aged ≥80 high-risk patients underwent definitive treatment.

Another frequently diagnosed cancer in older people is head and neck cancer. A large retrospective analysis of 14,909 oropharyngeal cancer cases from the Surveillance, Epidemiology, and End Results (SEER) programme assessed the extent of under treatment. Their results found that as age increased, the proportion of patients who did not receive any treatment significantly increased, whereas the number of patients who received a combined treatment approach also significantly decreased [9]. Only half of older patients with head and neck cancer are treated in accordance with standard guidelines and institutional protocols [10]. Likewise for lung cancer, patients over 70 years of age account for approximately 47% of lung cancer patients, and are less likely to receive curative treatment [11], [12]. The reasons for the lack of adherence to guidelines are unclear, and may well be related to disease and patient characteristics. However, better clarification and rationalisation of these treatment decisions in older adults are needed.

It is commonly accepted that chronological age is a poor predictor of treatment tolerance and outcomes in older adults [13], [14], and a more objective assessment of underlying frailty is needed. Intrinsic vulnerability of older adults with cancer and response to cancer-directed treatment, is a function of underlying frailty rather than chronological age [15]. Capturing this heterogeneity is often suboptimal in clinical practice, as frailty and associated terms remain poorly understood in radiation oncology [16], [17], [18], despite the fact that the majority of patients we see are older. In addition, the prevalence of frailty in newly diagnosed patients with cancer is known to be high, with over half of older patients categorised as frail or pre-frail (at risk of frailty) [19], [20].

In recent times, there has been much focus on the need for ”personalised treatment” in order to plan the most precise treatment for the individual person, for maximum therapeutic potential, with the lowest risk of toxicity [21]. This is often defined by cancer biology, and numerous predictive biomarkers have been discovered. However, one notable way to personalise cancer treatment in those who are older, is by capturing physiologic reserve capacity i.e. frailty. This review will focus on the definition of frailty, and the potential role of frailty assessment in clinical practice in order to enhance person-centred care.

## What is frailty?

Performance status, measured by the Eastern Cooperative Oncology Group (ECOG) or Karnofsky performance status measures, attempts to capture the functional status of the person with cancer, and is widely used in treatment decision making in radiation oncology. However, it is subjective, and does not reflect the full extent of vulnerability among older adults with cancer [22], [23]. Therefore, other more objective assessments are required.

To this end, the concept of frailty is an important one, and forms the basis for the practice of geriatric medicine, but remains poorly appreciated in other aspects of medicine, such as radiation oncology. It has been defined as a consequence of decline in many physiological systems, resulting in a reduced reserve capacity and increased vulnerability to stressors [24], [25]. This vulnerability, usually age-related, confers an inability to maintain homeostasis in the face of a physiological threat e.g. cancer treatment. Under normal conditions, human beings are able to withstand a certain amount of decline, without any great impact on everyday life. Nonetheless, when a major physiological stressor is introduced, such as a major illness like cancer, this might destabilise an otherwise well-functioning individual, and lead to a loss of resilience. This loss of resilience may mean the difference between being independent in the aftermath of treatment, or becoming dependent so that hospitalisation or full-time care are now necessary. The patient’s treatment decision may thus be impacted by such an outcome, as demonstrated in some studies to date [26], [27].

## How do we assess frailty?

The gold standard in terms of clinical assessment of frailty is Comprehensive Geriatric Assessment (CGA). CGA is noted to be time consuming however, and requires some degree of specialist training. Therefore frailty screening is a more feasible option in an already resource-constrained radiotherapy department. This two-step approach has been recommended by the National Comprehensive Cancer Network (NCCN) [28] the International Society of Geriatric Oncology (SIOG) [29], [30] and European Organisation for Research and Treatment of Cancer (EORTC) [31]. This involves the use of a short screening tool to identify those who would benefit from a full CGA, followed by administration of the CGA to those who screen positive.

### Frailty screening

In relation to screening, two schools of thought predominate in the gerontology literature, the phenotype of frailty defined by Fried [32], from the Cardiovascular Health Study (CHS), and Rockwood’s clinical frailty criteria [33], based on cumulative deficits on various CGA domains.

The frailty phenotype (FP) defined by Fried, is relatively short and easy to use. Its focus is on physical frailty, assessed using five components, including (1) unintended weight loss, (2) weakness (low grip strength), (3) exhaustion, (4) low physical activity and (5) slow gait speed [32]. This results in a categorisation as robust if no deficits are identified, prefrail if only 1–2 deficits are present, and frail if there are 3 or more. This may be very relevant in cancers where weight loss is a significant factor e.g. gastrointestinal cancers. However, frailty is also associated with weight gain, especially in the era of higher obesity rates [34]. This can also induce frailty, for example in hormone-dependent tumours like prostate cancer, where there is significant loss of muscle mass due to androgen deprivation, in those who are prescribed hormone therapy. This reduced muscle mass and quality is termed sarcopenia, and is commonly used as a surrogate marker for frailty [35]. Skeletal muscle wasting, that may be obscured within the bulk of body weight as patient’s age, is known as sarcopenic obesity [36]. Bylow et al., have highlighted this phenomenon in their study of 131 patients with prostate cancer, and replaced the weight loss item in the original FP, with weight gain [37]. Doing so was much more informative in terms of diagnosing frailty, and resultant risk of adverse outcomes e.g. falls, hospitalisations, toxicity and even death. Therefore, selection of frailty assessment tools may be individualised based on perceived need for a particular type of cancer, and those initially designed for the general population may not always suit oncology.

The second main frailty screening approach in geriatric medicine is Rockwood’s frailty index (FI). The original FI included 92 individual deficits from a wide range of domains (including cognitive, psychological and social factors), which were used to collectively define frailty [38]. Subsequent work reduced the number of FI items to 30 or so, with no resultant loss of validity [39], [40]. There is greater in-built redundancy in the FI approach, compared to the phenotypic model, as it includes a greater number of items. Therefore, three deficits will not render someone frail, as it does in the phenotypic approach, and a greater number are used in operationalising frailty using the FI. The range of domains included has been deemed more useful in a clinical setting, as it is widely known, especially in cancer care, that that these factors are important socio-environmental determinants of health and wellbeing. Deficits are defined as “any symptom, sign, disease, disability or laboratory abnormality that is associated with age and adverse outcomes, present in at least 1% of the population” and covers several organ systems [40], [41]. Typical examples include comorbid conditions (e.g. cardiovascular disease, diabetes osteoporosis), cognitive and mental health, visual impairment, as well as activities of daily living.

Also developed by Rockwood et al., the clinical frailty scale (CFS) is an even shorter (7 item) measure of frailty based on clinical judgement and the deficit accumulation approach mentioned earlier [33]. It is a well-established, quick and easy, scale to define frailty, and the most popular tool used in geriatric medicine in Canada and the UK, as well as in published research [42]. Its evaluation, thus far, in those who have cancer, is limited to one small surgical series of patients with pancreaticobiliary and melanoma cancers. In this study, the CFS was deemed to have greater discriminatory power than the more commonly used ECOG performance status [23]. A validation study in community-dwelling older people demonstrated that it was a better predictor of mortality than simple measures of cognition, function or comorbidity [33]. Its obvious advantage in a clinical setting is the relative ease of use, compared to other longer assessments of cumulative deficits, such as the original 70 item Frailty Index [43], to which it has correlated well, in terms of validity and reliability [44], [45].

Frailty is potentially reversible, when managed effectively and appropriate interventions put in place to deal with deficits identified [46], [47]. This has the potential to prevent falls, hospitalisations, nursing home placement and other important quality of life (QoL) indicators [48].

One of the most acceptable screening methods developed to date in the oncology literature is the Geriatric-8 or G8 [49]. Use of a screening tool, such as the G8 (Table 1), enables healthcare professionals to use (scarce) resources more effectively, while ensuring that patients receive optimum care. The G8 was the first screening tool devised specifically for oncology, and has been validated in the ONCODAGE study of patients with cancer [50]. It has shown high sensitivity (65–92%) and acceptable specificity, taking approximately 4 minutes to complete [51]. Poor performance on the G8 is associated with poorer one year survival [50]. A more recent systematic review of the G8, incorporating 46 studies, on the performance of the G8, have also found an association with survival and treatment-related complications [52]. A further development is a self-report version of the G8, with a preliminary analysis demonstrating good concordance with the original G8 [53]. VES-13 is another screening tool that has been used in oncology, and is largely based on functional status, but was not developed specifically for oncology [54].

**Table 1:** G8 screening tool with score indicating impairment.

Items	Possible answers	Score
Food intake in the last 3 months	**0:** severe reduction in food intake **1:** moderate reduction in food intake **2:** normal food intake	

Weight loss during the last 3 months	**0:** weight loss >3 kg **1:** does not know **2:** weight loss between 1 and 3 kg **3:** no weight loss	

Mobility	**0:** bed or chair bound **1:** able to get out of bed/chair but does not go out **2:** goes out	

Neuropsychological problems	**0:** severe dementia or depression **1:** mild dementia or depression **2:** no psychological problems	

Body Mass Index (BMI)	**0:** BMI < 19 **1:** BMI 19–<21 **2:** BMI 21–<23 **3:** BMI 23 or greater	

Takes more than 3 medications per day	**0:** yes **1:** no	

The patient’s self-rated health status (compared to other people of the same age)	**0:** not as good **0.5:** does not know **1:** as good **2:** better	

Age	**0:** >85 **1:** 80–85 **2:** <80	

Total score (0–17) [**Cut-off ≤ 14**]		

### Comprehensive Geriatric Assessment (CGA)

The gold standard for frailty assessment is Comprehensive Geriatric Assessment (CGA). CGA is a multidimensional, multidisciplinary assessment that includes functional status, comorbidity, cognition, nutritional status, social support, polypharmacy and psychological status, at a minimum [55]. Conducting this assessment, which can often take one hour or more, provides an indication of the accumulation of deficits in multiple domains, as mentioned previously. This can, in turn, provide a broader overall understanding of an individual’s health status, that affects their life expectancy, level of functional decline and cognitive decline. It incorporates patient’s own wishes, as well as how oncologic treatment might potentially affect them [56]. CGA has been proven to predict a range of outcomes from hospitalisations, dependence and ability to remain in one’s home [57]. The core domains of CGA are often interrelated e.g. cognitive decline may cause a reduction in physical activity and inability to manage basic care, such as feeding and medications. This, in turn, can lead to functional decline and falls, resulting in hospitalisations. CGA is often abbreviated to GA (geriatric assessment) in the geriatric oncology literature, representing a less comprehensive approach performed by the oncology team.

The following section provides a brief overview of how each domain might impact radiotherapy treatment.

#### Functional status

An assessment of functional status determines how fit the patient is for treatment. Objective measures of functional status include gait speed, balance, grip strength and lower extremity strength, which have been shown to be predictive of various patient outcomes [58]. In particular, gait speed is a significant predictor of mortality across numerous studies [59], and is a relatively simple assessment to complete. Subjective measures, include Activities of Daily Living (ADLs) and Instrumental Activities of Daily Living (IADLs). These provide information on the person’s ability to perform basic activities related to self-care (ADLs), or the ability to function independently in their communities (IADLs).

**Fig. 1:**
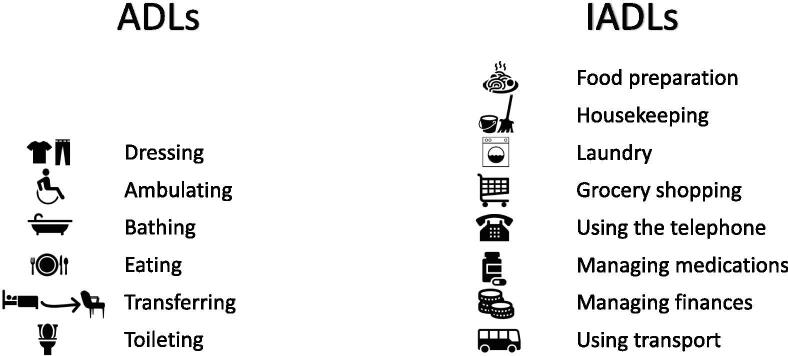
Components of Activities of Daily Living (ADLs) and Instrumental Activities of Daily Living (IADLs).

The individual components of each are shown in Fig. 1 below.

Mohile et al. [60], in a study of more than 900 patients with prostate cancer undergoing radiotherapy, found no difference in toxicity in older patients, but older patients were more likely to report that symptoms interfered with walking after radiotherapy. Again, this underlines the importance of assessing functional status in patients with cancer, and ensuring optimisation of mobility both during and after treatment, in order to prevent further decline. Such decline could increase the risk of falls, which, in conjunction with androgen deprivation therapy (ADT), is another age-related risk that is prevalent in those with cancer [61], [62].

#### Cognition

Cognitive assessment is another key component of CGA, and determines a patient’s ability to provide informed consent, as well as their fitness for treatment. In oncology, most research in this area, has been conducted on patients with breast cancer receiving chemotherapy, with or without hormone therapy [63], [64], or in prostate cancer, with patients undergoing ADT [65], [66]. Testosterone is known to have protective effects on cognition [67]. Therefore its depletion has important repercussions for patients, and is also linked to the development of frailty [68]. Assessing cognition is also important in order to diagnose dementia, which often goes unrecognised in the hospital setting [69]. Early diagnosis of cognitive impairment is important in order to implement earlier treatment and effective management. It is also necessary for delirium prevention, treatment modification and compliance monitoring.

#### Comorbidity

Comorbidity increases with age, and is associated with poorer overall survival in older adults with cancer [70]. Over two-thirds of older individuals have been shown to have two or more medical conditions, and almost a quarter have four or more [71]. These comorbidities include cardiovascular disease, diabetes and renal impairment, which can greatly increase the risk of complications from cancer-directed treatment. They also need to be considered as part of the overall risks and benefits of treatment, in collaboration with the patient [70]. Comorbidity also increases the likelihood of being prescribed more medications and therefore the subsequent potential for adverse drug reactions [72].

#### Nutrition

Malnutrition is relatively common alongside a diagnosis of cancer [73], [74]. Nutritional status and weight loss are known to predict complications from treatment and increased mortality in older people with cancer [75]. Poor nutrition also leads to things like osteoporosis and associated issues, including falls and fractures [76]. Especially relevant to radiotherapy is the increased risk of mucositis in older patients, particularly evident when treating cancers of the head and neck region [77]. Weight loss also has independent prognostic value in this group of patients [78], therefore ensuring adequate supportive care and dietetic support during radiotherapy is very important.

#### Social support

Receiving a diagnosis of cancer can greatly affect a person’s social activities, and a lack of social support has been linked to frailty as well as mortality [79]. Social support is critical for patients who are required to attend daily radiotherapy treatments. Many may find daily travel tiring, and struggle to navigate unfamiliar urban areas, in order to access care. This is one area where shorter fractionation schedules, as outlined below, are important. Considering shorter overall treatment schedules can lessen the overall impact on the patient, and minimise the stress associated with travel.

#### Polypharmacy

Polypharmacy is generally defined as the concomitant prescription of five or more medications, while excessive polypharmacy is categorised as 10 or more [80]. Naturally, as the number of comorbid conditions increases, so too does the risk of drug reactions from increasing polypharmacy [81]. Older adults with cancer frequently experience excessive polypharmacy, which in turn poses an enhanced risk of functional decline [82], falls and fractures [83], hospitalisations [84] as well as mortality [85]. Assessment of polypharmacy, as part of CGA, is therefore instrumental in avoiding or eliminating potentially inappropriate medications.

#### Psychological status

For older people diagnosed with cancer, there is much variation in the published literature in terms of prevalence, ranging from 15 to 30% [86], [87]. Depressive symptoms can greatly affect QoL and lead to functional decline and social isolation, if not detected and managed [88]. This can lead to frailty [89], and is also associated with declining cognitive function [90].

### Personalised radiotherapy: The role of frailty assessment

The evidence base for frailty assessment in radiation oncology is particularly poor. Twelve non-randomised studies were included in a recent systematic review by Szumacher et al. [91]. Of these, four studies used a screening tool alone, while the remainder used the recommended combined approach of screening, followed by CGA. Only two studies showed a significant association between screening and mortality outcomes, while only one demonstrated that CGA had an influence on treatment decisions. Half of included studies found an association between screening or CGA, and treatment tolerance. The majority of these studies included small sample sizes. However, there is an indication as to how CGA might be useful in radiation oncology, which merits further research.

To that end, studies are ongoing which will almost certainly influence clinical practice in the future. The focus is now moving towards the role of CGA-driven interventions, like the RCT by Soubeyran et al. [92], who are investigating these interventions and associated therapeutic outcomes. This is based on an initial screening with the G8, and includes patients referred for radiotherapy. A further example, the multicentre ELAN trial [93] aims to stratify patients according to CGA-based allocation, and select treatment accordingly. Frail patients will be randomised to the ELAN-RT arm, which is a hypofractionated split course schedule delivering 30 Gy/10 fractions, followed by 25 Gy/10 fractions after a two-week gap for recovery.

Identification of previously unknown deficits is one of the major advantages of frailty screening and CGA, allowing some intervention in order to optimise patient care and potentially reverse frailty. A limited number of other, non-randomised, studies, have been conducted in radiation oncology. Notwithstanding the limitations imposed by research design, many of these studies have indicated that CGA can influence care. Goineau et al. [94], in a study of patients (n = 100; ≥75 years) with localised prostate cancer, undergoing radiotherapy treatment, found no association between CGA and QoL. However, they discovered IADL impairments at baseline in half of study participants, as well as ADL impairments in 16% of patients. About 20% presented with cognitive decline, 31% with depressive symptoms and more than two-thirds with major co-morbidities. Malnutrition was virtually absent, again suggesting that frailty measures based on weight loss would have little relevance. Spyropoulou et al. [95], in a radiotherapy patient population (n = 230) found that patients >75 years with higher Vulnerable Elders Survey-13 (VES-13) [96] scores were less likely to complete radiotherapy, independent of other factors that might affect radiotherapy completion. Neve et al. [97], in a further small study of older patients with head and neck cancer, also receiving radiotherapy, found that patients identified as vulnerable at baseline, were less likely to complete radiotherapy.

These studies signal some of the potentially useful interventions for patients receiving radiotherapy, albeit not directly referenced in most studies to date, which have focused exclusively on assessment, without mention of follow-up care. This area has been one of the gaps in the current literature in oncology generally, but more so in radiation oncology. Table 2 below outlines some of the possible interventions that may be used for each CGA domain/deficit.

**Table 2:** CGA-driven interventions in oncology [adapted from ASCO guidelines [98]].

CGA Domain in Which Deficit Lies	Possible CGA Driven Interventions
Functional status	Physiotherapy and/or occupational therapy referrals for strength and balance training, home safety evaluation, exercise prescription
Comorbidity and Polypharmacy	Involve General Practitioner and/or geriatrician in decision making and disease specialists for management of comorbidities, review medications and eliminate redundant/unnecessary medications, consider pharmacist review, assess adherence to medications
Cognition	Assess decision-making capacity and ability to consent to treatment, identify healthcare proxy and involve proxy in decision making for treatment, assess delirium risk and counsel patient and family, undertake medication review to minimise medications with a high risk of delirium, consider geriatrician referral
Depression	Consider referral to psychotherapy/psychiatry/psycho-oncology, cognitive behavioural therapy, social work involvement and pharmacologic treatment
Nutrition	Dietician referral and nutrition counselling, assess need for additional support for meal preparation and home support interventions

Some of the ways in which CGA might alter treatment decisions in radiation oncology include the omission of concomitant/neoadjuvant chemotherapy or surgery for example, which can contribute considerable toxicity for the patient. Another adaptation is altering the type and modality of radiation offered to patients. Although radiotherapy is usually well tolerated in older patients [60], hypofractionated regimes should be considered in those with poor support structures, poor mobility, transportation issues, in geographically remote areas, or in active caregiver roles. Hypofractionation is advisable in order to limit the burden of travel for such patients. There are many examples of its use in those who are older/ frail, many of which were adopted during the COVID pandemic [99], [100] e.g. glioblastoma multiforme (GBM). A treatment regime of 25 Gy in 5 treatments has been shown to be non-inferior to 40 Gy in 15 treatments [101]. Other examples include the use of short course RT (1 week) over long course chemoradiation in locally advanced rectal cancer [102], the FAST-forward regime, delivered over one week, for early breast cancer [103] and moderate hypofractionation in prostate cancer with the CHHiP protocol [104], with a recommended dose adjustment for those aged >75 years [105]. These are now proven to be efficacious, and indeed standard practice, for many sites, for all patients, but are underutilised.

Indeed, many of the adaptations that may be made for older patients with coexisting cancer and frailty, have recently been highlighted as recommendations in the era of the COVID-19 pandemic [106]. These include facilitating telephone or telemedicine consultations, which are feasible in numerous cancer sites [107], [108], [109]. These types of remote consultations are particularly suitable for older patients with social frailty, and associated difficulties related to travel for radiotherapy. Remote consultations avoid unnecessary travel, without compromising patient care, however it is known that older people may have less access to technology and more challenges in relation to digital literacy [110].

For palliative radiotherapy, treatment courses should also be kept as short as possible for those who are frail. Reduction of regimes to single-fraction, where appropriate, for example in the management of bone metastases is preferable, known to be equally effective, compared to longer regimes [111], [112], [113]. Likewise, for patients with brain metastases, shorter regimes have similarly shown equivalence [114].

Other radiotherapeutic adaptations may be deemed suitable for older patients, such as the use of advanced technologies to minimise toxicity. An example of this is the use of stereotactic radiosurgery (SRS) alone in brain metastases, compared to SRS combined with whole brain radiation, which may provide better cognitive outcomes [115]. Techniques such as volumetric arc therapy (VMAT), can also greatly assist older adults with mobility restrictions or movement disorders e.g. Parkinson’s disease, as they reduce time on the treatment couch.

Many site-specific recommendations, including the complete omission of radiotherapy, where appropriate, are made in the aforementioned COVID-19 response paper by Simcock et al. One example of this is in the treatment of frail patients with Glioblastoma Multiforme (GBM), as mentioned previously [101]. Alternatively, in sites such as lung cancer, CGA may help to identify frail patients who are not candidates for conventional, daily radiotherapy but may benefit from other (curative) modalities, such as stereotactic body radiotherapy, with fewer hospital visits and potentially less toxicity [116]. Accelerated Partial Breast Irradiation (APBI) is another option to simultaneously limit toxicity and afford greater convenience for the patient [117]. APBI uses larger radiation doses to the localised tumour bed (as opposed to the entire breast) over a shorter period of time.

### Implementation in clinical practice

**Fig. 2:**
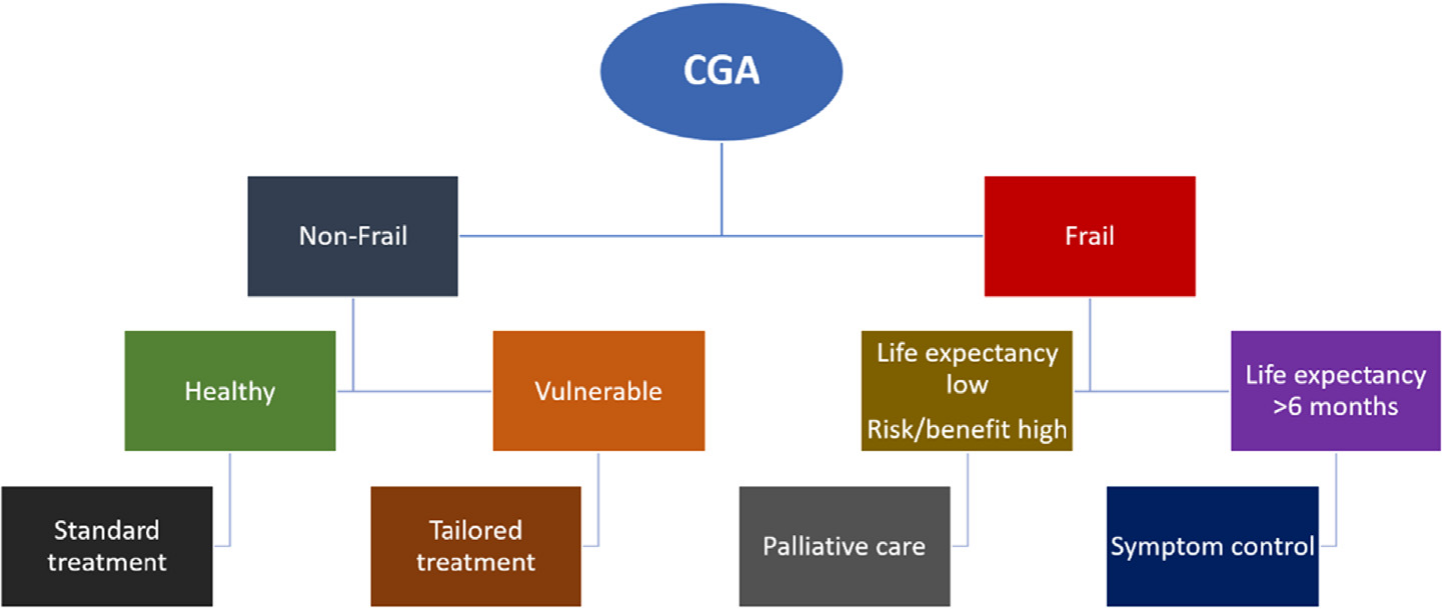
Conceptual model of how CGA can be incorporated into oncology assessment and treatment.

Geriatric oncology does not exist in many radiotherapy departments at the current time, but there are many international models to use as exemplars as to how it may be implemented in clinical practice [118], [119], [120]. One major challenge is that there is a notable shortage of geriatricians worldwide [121]. However, international models of geriatric oncology are based upon upskilling oncologists, nurses and allied health professionals to be able to implement, understand and interpret the findings of a CGA and how they may impact patient care [122]. Patient and/or caregiver self-report is also feasible for many of the domains of CGA, including electronic methods [53]. A two-step model with a brief initial screening, followed by full assessment allows a better allocation of resources in the oncology setting [118]. Fig. 2 depicts a conceptual model of how CGA can be incorporated into oncology assessment and treatment. Fit patients should be candidates for the same treatment as their younger counterparts, while frail patients would benefit from a more palliative approach. Vulnerable patients may need to be offered a tailored treatment in order to avoid decline during/after treatment, or may benefit from a dose adapted approach.

Estimates of the total time required for a basic assessment in oncology range from 22 to 27 minutes in total [119], [123], with the healthcare professional present for only a fraction of that time [124]. In terms of cost-effectiveness, the relative cost of CGA is small compared to the various diagnostic tests and scans that are used in oncology [125].

Other members of allied health professional groups have also been identified as key contributors to the multidisciplinary team in geriatric oncology [126]. Occupational therapists and physiotherapists are uniquely positioned to provide supportive services for patients in danger of functional decline. Dietician collaboration is essential for nutritional issues. Pharmacists can provide much needed insight into polypharmacy and potentially inappropriate prescribing, while psycho-oncology services and social workers can assist with psychological or social issues. Harnessing the skills and expertise of the existing multidisciplinary team is essential in geriatric oncology. Not every department will have access to the full array of specialists, but it is important to remember that CGA and screening can be provided by physicians, nurses and any other healthcare professional. There is great potential to expand the current RTT role in this context. A Canadian study of RTT’s opinions on the their role in specialised geriatric oncology clinics [127], has identified the need for such role development, and this remains an unexplored and exciting avenue for future studies.

There are many models of geriatric oncology programmes, such as those providing ongoing geriatric oncology management throughout the cancer trajectory, one-time consult programmes, site specific models and those based on age, rather than tumour site [128]. France has one of the most coordinated systems of geriatric oncology in Europe, and serves as one of the exemplars worldwide. This coordination has been facilitated by funding through the Institute National du Cancer (INCa), who have supported consecutive cancer plans [129]. This has led to a more coordinated network of geriatric oncology units across the country, which are led by both an oncologist and a geriatrician. Ultimately, this has enhanced access for older patients and resulted in organised geriatric oncology research programmes and increased awareness among both the general population and health professionals.

The Italian system of geriatric oncology is coordinated through The Italian Geriatric Oncology Group (GIOGER) [130], which is similar to the Spanish system [131], with a few geriatric oncology programmes in some of the biggest centres. Likewise, in the UK, a number of pilot programmes have been initiated [132]. The use of a frailty screening tool is a quality indicator for patients with colorectal cancer in the Netherlands [133].

A geriatric oncology programme requires clinical and research infrastructure, as well as administrative support to lay the foundations for a sustainable programme in the longer term. Difficulties sustaining these requirements have been explored in the published literature [134]. Defining clinical referral pathways for identified deficits and ensuring access to appropriate interventions are important tasks to address before implementing CGA [135]. This requires good communication with other disciplines as part of the multidisciplinary pathway, especially with geriatric medicine colleagues, which has historically been quite poor, with both professions traditionally working with little collaboration [136]. General Practitioners (GPs) are another untapped resource that could potentially be better utilised in geriatric oncology. GPs often feel excluded during cancer treatment, despite being a main point of contact for the patient, and often the best gatekeeper for access to support services in the local community [137], [138].

Bagayogo et al. [139] outlined ways in which oncologist-geriatrician collaboration could be enhanced, such as institutions mandating the presence of the geriatrician at multidisciplinary meetings, or tumour boards. Oncologists indicated that this would be useful, and also that physical proximity of geriatricians would be ideal. Health technology was also identified as a good facilitator of communication and collaboration [136], and having “geriatric oncology champions” in academic oncology [140]. Four recent pilot studies (three RCTs and one cohort study) examined the role of a multidisciplinary collaborative approach to CGA and associated interventions in oncology [141], [142], [143], [144]. These studies have demonstrated a positive impact from a multidisciplinary geriatric oncology team with regard to patient outcomes, such as QoL.

To implement CGA into clinical practice, there are also some educational requirements in the medical, nursing and allied health curricula that need to be addressed. There is an unmet need in this regard [16], [17]. In order to address this, efforts to devise a core curriculum in geriatric oncology have been undertaken by several societies. ASCO [98] and the European Society of Medical Oncology (ESMO) [145] have both developed recommendations for geriatric oncology as a part of their global curricula. Likewise, the European Oncology Nursing Society (EONS) has also published recommendations for a core curriculum for geriatric oncology for the nursing profession [146]. Similar efforts are underway in radiation oncology [147].

## Conclusion

In the era of personalised treatment, there is a growing need for implementation of frailty assessment in clinical practice, in order to stratify care, as well as better collaboration with geriatric medicine colleagues. CGA is a multidimensional assessment used to assess an older patient’s cognitive function, co-morbidities, physical function, psychological function, nutritional status and the patient’s social support system. It allows oncologists to have a better estimation of the patient’s overall health status i.e. is the patient fit, vulnerable or frail? This can potentially inform treatment decision making and allow a more patient-centred process of care. There are many international models of geriatric oncology, from which we can learn, in order to ensure implementation is successful. This is recommended by best practice guidelines and offers opportunities for clinical practice and research, as well as professional role development.

## Declaration of Competing Interest

The authors declare that they have no known competing financial interests or personal relationships that could have appeared to influence the work reported in this paper.
